# Anthropometric Determinants of Rowing Performance in a Multinational Youth Cohort

**DOI:** 10.3390/jfmk11010039

**Published:** 2026-01-17

**Authors:** László Suszter, Zoltán Gombos, Ottó Benczenleitner, Ferenc Ihász, Zoltán Alföldi

**Affiliations:** 1 Sport and Health Sciences Research Group, Eszterházy Károly Catholic University, 3300 Eger, Hungary; gombos.zoltan@uni-eszterhazy.hu (Z.G.); benczenleitner.otto@uni-eszterhazy.hu (O.B.); 2 Department of Health Promotion and Exercise Science, Széchenyi István University, 9021 Győr, Hungary; ihasz.ferenc@sze.hu (F.I.); alfoldi.zoltan@sze.hu (Z.A.)

**Keywords:** anthropometry, rowing performance, talent identification

## Abstract

**Background:** Rowing performance in youth athletes is strongly influenced by anthropometric characteristics, body composition, and limb proportions; however, the combined contribution of these factors across developmental stages remains insufficiently understood. This study investigated the relationships between key anthropometric variables and ergometer performance in a multinational cohort of young rowers. **Methods:** A total of 194 athletes (48 females, 146 males) from ten countries participated. Based on age and sex, participants were categorized into junior female (JF), junior male (JM), adult female (AF), and adult male (AM) groups. Body height, body mass, body fat (F%), relative muscle mass (M%), limb lengths, and body surface area (BSA) were measured. Rowing performance was assessed via maximal 2000 m ergometer trials. **Results:** Males outperformed females across all age groups (*p* < 0.001). Performance showed strong positive correlations with body height (r = 0.673, *p* = 0.003), body mass (r = 0.724, *p* = 0.005), arm span (r = 0.681, *p* = 0.002), lower-limb length (r = 0.394, *p* = 0.004), relative muscle mass (39.9 ± 5.2%; r = 0.531, *p* < 0.001), and especially BSA (1.94 ± 0.19 m^2^; r = 0.739, *p* < 0.001). Relative body fat was negatively associated with performance (17.6 ± 6.9%; r = −0.465, *p* < 0.001). **Conclusions:** Findings indicate that rowing performance in youth athletes reflects multidimensional anthropometric configurations rather than isolated traits, characterized primarily by the combined contribution of body surface area, relative muscle mass, and segmental body dimensions. From a practical perspective, higher-performing athletes typically exhibited body surface area values approaching or exceeding ~1.90 m^2^ and relative muscle mass above ~40%, suggesting these ranges as indicative reference benchmarks rather than fixed selection thresholds. Integrating anthropometric profiling with physiological assessment may enhance early talent identification and support individualized training strategies in competitive youth rowing.

## 1. Introduction

In performance-oriented sports such as rowing, evaluating athletes’ physical attributes and their relationship to athletic output is of critical importance. Anthropometric characteristics and physiological capacities—especially in young, developing athletes—play a key role in forecasting success and informing talent development strategies [1,2]. Recent research emphasizes that anthropometric and physiological parameters play a fundamental role not only in rowing but across various sports disciplines, highlighting their universal significance in performance evaluation and talent selection [3,4,5,6].

Rowing is unique among endurance sports in that the power is generated from a seated position. This distinguishes it from sports like running, where body mass is a limiting factor. In rowing, greater body mass is not inherently disadvantageous, and certain body proportions—such as long limbs, large body surface area (BSA), and extended arm span—can offer distinct biomechanical advantages by increasing stroke length and improving force application on the oar [7,8,9]. Different rowing styles, such as sweep and sculling, also exhibit characteristic anthropometric profiles [10].

Successful rowers tend to be taller, have longer limbs, and exhibit higher muscle mass and lower fat percentage compared to their less successful peers [11,12]. These traits enhance the effectiveness of the rowing stroke by maximizing mechanical efficiency and stroke power [13]. Numerous studies have found that height, body weight, and especially arm and leg length are strong predictors of performance on the rowing ergometer [14,15,16].

Anthropometric data are especially relevant in youth athletes, whose physical development is ongoing. Measurements taken during this dynamic growth phase may help predict long-term performance potential and support evidence-based selection processes [17]. The most commonly studied parameters include height, arm span, sitting height, leg length, and calculated indicators such as BSA [18,19].

However, rowing performance depends not only on morphology but also on metabolic capacity. A 2000 m rowing race demands both a highly developed aerobic system and robust anaerobic capacity. Depending on boat class and conditions, race duration ranges from 5.5 to 7 min, requiring intense effort from both energy systems [20]. While aerobic metabolism dominates the middle of the race, anaerobic contribution is crucial at the start and finish phases [21].

Maximal oxygen uptake (VO_2_max) is one of the most critical indicators of endurance capacity. Among elite rowers, VO_2_max often exceeds 6.5–7 L/min, supported by large lung volume and significant cardiovascular adaptations [22,23]. Pulmonary vital capacities of up to 9 L have been reported, significantly above average for athletes of comparable body size [24]. In parallel, stroke volume may reach up to 195 mL per heartbeat, and cardiac output during maximum exertion may approach 40–49 L/min [25]. Physiological demands are further evidenced by dramatic changes in blood pressure during rowing strokes. Due to Valsalva-like breathing maneuvers, systolic pressure may rise to 200 mmHg [26]. The extreme cardiorespiratory and ventilatory demands of rowing reflect the sport’s complexity and its requirement for whole-body physiological integration. Several studies highlight differing perspectives on the anthropometric determinants of rowing performance.

Several studies highlight the complex interplay between anthropometric and physiological factors in determining rowing performance. While some authors identify lean body mass as the strongest predictor, others emphasize overall body dimensions, proportionality, and limb leverage as key determinants of performance variability [27,28]. Rather than treating these perspectives as mutually exclusive, the present study adopts an integrative approach, examining whether rowing performance in youth athletes is better explained by isolated anthropometric traits or by multidimensional configurations reflecting the combined contribution of body size, body composition, and segmental dimensions.

Based on the existing literature and the developmental characteristics of youth rowers, we hypothesized that: (1) rowing ergometer performance would be positively associated with body surface area, relative muscle mass, and segmental body dimensions, and negatively associated with relative body fat; (2) body surface area would demonstrate a stronger association with performance than individual body composition indices alone; and (3) performance variability would be better explained by the combined contribution of multiple anthropometric characteristics than by any single isolated trait.

## 2. Materials and Methods

A total of 194 young rowers from ten countries participated in this study, including 48 females (mean age: 18.7 ± 3.1 years) and 146 males (mean age: 19.3 ± 4.2 years). The sample included athletes from Slovenia (n = 21), Germany (n = 9), Croatia (n = 47), Hungary (n = 86), Serbia (n = 22), Portugal (n = 1), Austria (n = 2), Bulgaria (n = 3), Slovakia (n = 2), and the Czech Republic (n = 1). For the purposes of the analysis, participants were stratified into four age–sex groups. Athletes aged 16–18 years were categorized as junior females (JF, n = 27) and junior males (JM, n = 71), whereas those older than 18 years were classified as adult females (AF, n = 21) and adult males (AM, n = 75). Although biological maturation is recognized as an important factor in studies involving developing athletes, direct indices of biological maturation (e.g., years from peak height velocity or maturity offset) were not assessed in the present study. This decision was based on the characteristics of the sample, which consisted exclusively of nationally elite junior and adult rowers competing in internationally recognized age categories. As reflected by the absence of substantial differences in key anthropometric variables between junior and adult groups, the cohort can be considered relatively homogeneous with respect to maturational status. Therefore, biological maturation was not expected to represent a primary source of variability in the present analyses.

Participants were recruited using a convenience sampling strategy during an international rowing competition, in cooperation with national rowing federations and team coaches. All eligible athletes competing in the respective age categories were invited to participate, resulting in a high response rate. The applied age stratification (16–18 years vs. >18 years) reflects internationally recognized competitive rowing categories corresponding to junior (U19) and adult levels, allowing for developmentally and competitively meaningful comparisons. Only athletes classified within the national elite level of their respective age group were included in the study, while those who had experienced illness or injury in the month preceding the measurements and were therefore unable to participate in regular training were excluded.

Data collection took place during an international rowing competition in Bled and was conducted in full accordance with the ethical principles of the Declaration of Helsinki. All procedures followed the guidelines and regulations of the Scientific and Research Ethics Committee of Széchenyi István University (approval number: SZE/ETT-46/2025; approved on 30 July 2025). Participation was voluntary, and both athletes and their legal guardians received comprehensive information about the study prior to providing written informed consent. The research was conducted in cooperation with the participating sports clubs and national rowing federations.

### 2.1. Anthropometric Measurements

Anthropometric data were collected using certified Sieber-Hegner measuring instruments, following the guidelines of the International Biological Program [29]. A trained anthropometrist took all the anthropometric measurements in accordance with the standardized procedures of the International Society for the Advancement of Kinanthropometry (ISAK, Level 1), [30]. All anthropometric measurements were performed by ISAK-certified anthropometrists following standardized ISAK protocols. To ensure measurement reliability, repeated measurements were conducted in accordance with ISAK guidelines, and technical error of measurement (TEM) was monitored throughout the data collection process. The obtained TEM values were within the acceptable limits recommended by ISAK for both basic and derived anthropometric variables, indicating high inter-rater and intra-rater reliability. The following parameters were recorded:

Body height (BH) and body weight (BW) were measured with participants barefoot and wearing light clothing.

Sitting height (SH) was defined as the vertical distance between the vertex (the highest point of the head) and the horizontal seating surface. Participants were required to sit upright on a firm, flat surface with the thighs and lower legs forming a 90° angle, the knees touching the seat edge. The trunk was held erect with the spine maximally extended, and the head aligned according to the Frankfurt horizontal plane.

Arm span (AS) was measured with arms extended horizontally at shoulder level (palms facing forward). The distance between the tips of the middle fingers was recorded. The examiner maintained the anthropometer at clavicle height, ensuring a horizontal plane.

Body mass index (BMI) was calculated using the Quetelet formula: BMI = BW (kg)/BH^2^ (m^2^). Body surface area (BSA) was calculated using the Mosteller formula: BSA (m^2^) = √[(BH in cm × BW in kg)/3600].

### 2.2. Body Composition

Body composition and its components (e.g., fat mass, lean mass, and total body water) were assessed using a multi-frequency bioelectrical impedance analysis (BIA) device (InBody 220, Biospace Co. Inc., Seoul, Republic of Korea) [31]. The device operates on the principle of bioelectrical impedance analysis and utilizes a tetrapolar eight-point tactile electrode system with foot-to-foot, hand-to-hand, and hand-to-foot contact, enabled by two stainless steel footplate electrodes and two hand-held electrodes.

Measurements were performed in accordance with the manufacturer’s standardized protocol and conducted during the morning hours. Participants were instructed to refrain from eating or drinking for at least 3 h prior to testing, to avoid strenuous physical activity on the day of measurement, and to maintain normal hydration status. Female participants were measured outside the menstrual phase whenever feasible.

The reliability of bioelectrical impedance analysis has been demonstrated previously in comparison with other body composition assessment methods, such as dual-energy X-ray absorptiometry (DXA) [32]. Relative muscle mass and body fat percentage were estimated using proprietary, population-specific prediction equations provided by the manufacturer.

### 2.3. Performance Testing

Rowing performance was assessed using a Concept2 Model D rowing ergometer through a maximal 2000 m time-trial test, during which average power output (W) was recorded. This ergometer is widely used in competitive and research settings and is considered a gold-standard instrument for off-water rowing performance evaluation [33]. Rowing performance was assessed using a standardized ergometer protocol. Prior to testing, all athletes completed a standardized warm-up consisting of light rowing at a self-selected intensity. Participants were familiar with ergometer testing as part of their regular training routines, and no additional familiarization trials were required.

Athletes were instructed to perform the test using an all-out pacing strategy, aiming to achieve maximal average power output over the test duration. All ergometer tests were conducted under standardized conditions, and the anthropometric assessments and performance testing were performed on the same day, with a standardized time interval separating the two procedures to minimize fatigue effects.

### 2.4. Statistical Analysis

Statistical analyses were performed using JASP (Version 0.16.0.0; JASP Team, University of Amsterdam, Amsterdam, The Netherlands). The distribution of each variable was examined using the Shapiro–Wilk test, and the homogeneity of variances was evaluated with Levene’s test. To assess sex-related differences, F-tests for equality of variances were conducted prior to group comparisons. As no substantial violations of normality or homogeneity assumptions were detected, two-sample Student’s *t*-tests were applied to compare male and female athletes, with the level of statistical significance set at *p* < 0.05.

To examine combined effects of age group (junior vs. adult) and sex, two-way analysis of variance (ANOVA) was performed for anthropometric and performance variables. When significant main effects or interactions were identified, post hoc pairwise comparisons were conducted using Bonferroni-adjusted tests to control for multiple comparisons. For exploratory age-related analyses within each sex, one-way ANOVAs were additionally applied using the predefined age categories.

Pearson’s product–moment correlation coefficients were calculated to assess associations between anthropometric characteristics, body composition variables, and rowing ergometer performance. Statistical significance for correlation analyses was set at *p* < 0.05.

## 3. Results

The difference in the mean heights (BH; cm), body weight (BW; kg), body fat (F%; %), body muscle (M%; %), arm span (cm), body surface area (BSA; m^2^), lower limb length (cm) and maximum power (Ergo; W) was significant between the male and female group (*p* < 0.05), the results and standard deviations of the two groups are shown in Table 1.

Table 2 presents the anthropometric and performance characteristics across age groups and sex categories, comparing junior female (JF), junior male (JM), adult female (AF), and adult male (AM) athletes. For most anthropometric variables, males (JM, AM) showed significantly higher values than their female counterparts (JF, AF), including body height, body mass, muscle mass, body surface area, sitting height, arm span, and limb length (*p* < 0.001). Relative fat mass showed the opposite pattern, with higher values observed in the female groups. Significant differences were also found in maximal ergometer performance: adult males achieved the highest values, followed by junior males, whereas both female groups showed lower performance levels (*p* < 0.001). Overall, the table highlights the strong influence of both age category and sex on anthropometric characteristics and rowing performance.

Significant main effects of age and sex were observed for body size, body composition, and ergometer performance variables (Table 2), whereas BMI did not differ significantly between groups.

**Figure 1:**
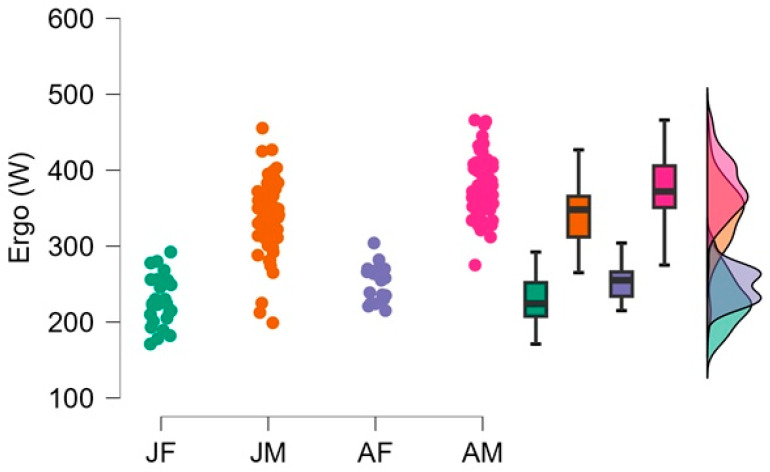
Age- and sex-related differences in maximal rowing ergometer power output; Legend: Colors indicate the different age–sex groups: JF = junior female, JM = junior male, AF = adult female, AM = adult male, JM, AM > JF, AF; AM > JM; *p* < 0.001.

Figure 1 illustrates the distribution of ergometer performance (Ergo, W) across the four age–sex groups (JF, JM, AF, AM). A distinct performance hierarchy is observable: adult males achieve the highest power outputs, with a narrow distribution indicating relatively homogenous high-level performance. Junior males follow with moderately high but more variable results. Female athletes form two clearly lower-performing clusters, with adult females outperforming junior females but still showing markedly reduced maximal power compared to both male groups. The accompanying boxplots and density distributions further emphasize the pronounced separation between sexes and age categories, reflecting the strong influence of morphological and developmental factors on rowing performance (Figure 1).

To identify which anthropometric variables most strongly influence performance, correlation analyses were conducted between performance (Ergo, W) and the principal body-size and body-composition parameters. The results showed distinct positive and negative associations: [Body height (cm; r = 0.673; *p* = 0.003); body mass (kg; r = 0.724; *p* = 0.005); arm span (cm; r = 0.681; *p* = 0.002); lower limb length (cm; r = 0.394; *p* = 0.004); sitting height (cm; r = 0.539; *p* = 0.002); relative muscle mass (%; r = 0.531; *p* < 0.001) and body surface area (BSA; m^2^; r = 0.739; *p* < 0.001)]. In contrast, a negative correlation was found between relative body fat and performance (F%; r = −0.465; *p* < 0.001).

**Figure 2:**
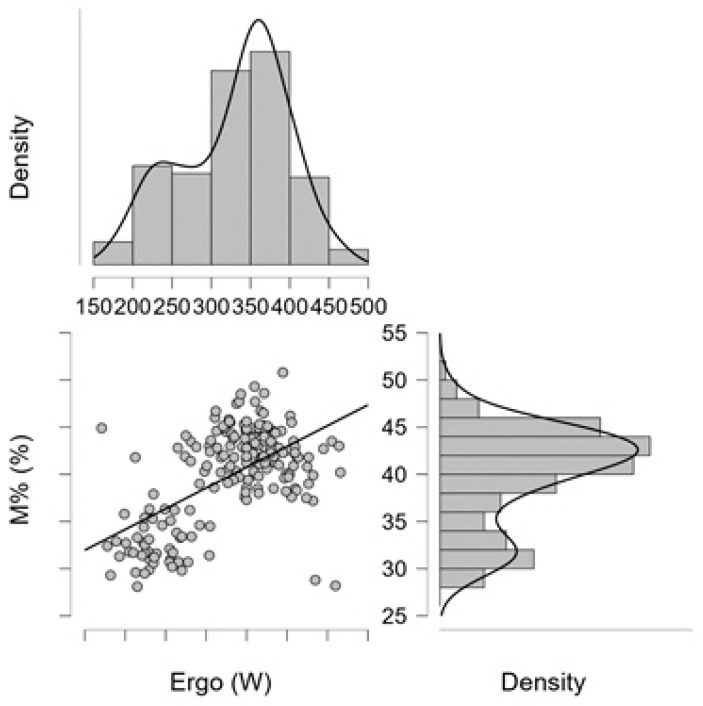
Relationship between performance (Ergo; W); and relative muscle mass (M%), (mixed-sex sample).; Legend: The horizontal axis shows the relationship between performance (W) and relative muscle mass (M%; %). The solid line serves as a visual indicator of the direction of the association and does not represent a fitted regression model. The marginal density plots illustrate the distributions of the variables [Ergo (W): M%, r = 0.531, (*p* < 0.001)].

Figure 2 displays the correlation between performance (Ergo, W) and relative muscle mass (M%), (r = 0.531; *p* < 0.001). The scatterplot reveals a clear positive relationship, whereby athletes with higher muscle mass percentages tend to achieve higher performance values. The line drawn across the data points serves as a visual trend indicator and does not represent a fitted regression model. The marginal histograms and density curves show that both variables exhibit unimodal but moderately skewed distributions, with performance clustering between approximately 250–400 W, whereas M% ranges primarily between 35 and 45%. Together, these distributions and the observed linear pattern suggest that muscularity is a substantial contributor to performance variability among the athletes.

**Figure 3:**
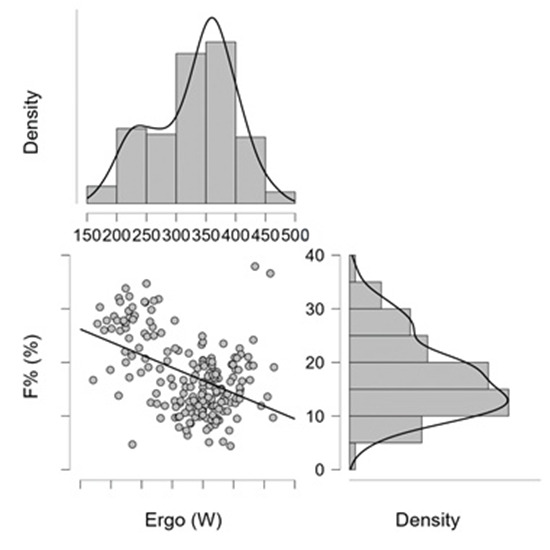
Relationship between performance (Ergo; W); and relative body fat (F%), (mixed-sex sample). Legend: The horizontal axis shows the relationship between performance (W) and relative body fat percentage (F%; %). The line indicates the direction of the negative trend, while the marginal density plots illustrate the distributions of the variables [Ergo (W): F%, r = −0.465, (*p* < 0.001)].

Figure 3 illustrates the correlation between performance (Ergo, W) and relative body fat percentage (F%), (r = −0.465; *p* < 0.001). The scatterplot indicates a clear negative association, whereby higher F% values generally correspond to lower performance outcomes. The line drawn across the data points serves solely as a visual trend indicator, highlighting the overall downward pattern rather than representing a fitted regression model. The marginal density plots display the distribution of each variable separately, with body fat percentage showing right-skewness and performance clustering around mid-range values. Together, these patterns support the observed inverse relationship between adiposity and performance.

For the total sample, mean (±SD) values were 1.94 ± 0.19 m^2^ for body surface area, 39.9 ± 5.2% for relative muscle mass, and 17.6 ± 6.9% for relative body fat.

## 4. Discussion

In our study, gender differences were evident, with male athletes demonstrating higher performance outcomes. Across all age and sex groups, increasing anthropometric values were associated with higher ergometer performance (W). Given that approximately 46.4 ± 4.5% of rowing force is generated by the lower limbs, 30.9 ± 5.2% by the trunk, and 22.7 ± 5.2% by the upper limbs, these associations are biomechanically plausible [34]. Accordingly, power–endurance capacities of the lower limbs—and, to a lesser extent, the trunk and upper limbs—are closely linked to rowing performance [28,35]. These findings align with previous work highlighting the combined contribution of lower-limb and trunk musculature to stroke efficiency and force production, underscoring the close interplay between anthropometric and physiological determinants of performance [35,36].

Beyond these expected sex- and age-related differences, the multivariate association patterns observed in the present study suggest that rowing performance cannot be explained by isolated anthropometric traits alone. Instead, performance appears to reflect characteristic combinations of body size, muscularity, and body composition [37,38,39]. In this context, the anthropometric–performance profiles discussed herein should be interpreted as conceptual and hypothesis-generating frameworks derived from descriptive and correlational patterns, rather than as empirically defined or statistically validated classifications. Future studies employing cluster or classification approaches are required to formally test and refine these proposed profiles [40].

Significant relationships between height, body mass, and performance were observed in both female and male athletes, consistent with previous reports [10,11,27]. Studies in adult rowers similarly emphasize the role of body mass [34], as well as absolute body size and proportionality, in achieving international-level success [14,27,28]. This likely explains why, in the present sample, greater stature and mass were associated with superior ergometer performance. Longitudinal observations further indicate that growth-related changes in body dimensions and composition directly affect technical efficiency and energy expenditure during youth development [41]. Prior work likewise demonstrates that larger rowers tend to perform proportionally better on the ergometer [21,26,27], and that body height and body surface area may represent some of the strongest predictors of output [25]. Collectively, these findings suggest that more successful rowers tend to be taller and heavier than their less successful peers [8], in apparent contrast to studies emphasizing lean body mass as the principal anthropometric correlate of performance [10,21,26].

Rather than representing a true contradiction, these perspectives may reflect different manifestations of the same underlying construct. Total body mass and body surface area likely act as proxies for the functional muscle mass available for force production, particularly in well-trained rowing populations. In contrast, body mass index did not emerge as a significant predictor of performance in the present study (Table 1 and Table 2). This likely reflects the limited sensitivity of BMI to distinguish between fat mass and lean mass or to capture sport-specific body proportionality in trained athletic populations. Together, these findings indicate that successful youth rowers tend to share a characteristic morphological pattern defined by the co-occurrence of larger body size, greater muscularity, and favorable segmental dimensions, rather than excelling due to any single isolated anthropometric attribute.

In addition to overall stature, the observed association between sitting height and rowing performance highlights the mechanistic relevance of trunk length in rowing biomechanics. Greater sitting height may confer performance advantages by increasing effective stroke length, enhancing force transmission from the lower limbs through the trunk, and supporting more efficient power transfer during the drive phase. This finding reinforces the importance of segmental body dimensions, beyond total body height alone, in explaining performance variability among competitive rowers.

Interpretation of absolute power output should nonetheless consider body-size dependency. Although allometric scaling approaches (e.g., allometric scaling of power output expressed as W·kg^b, where b denotes the allometric exponent, or normalization to body surface area) were not applied in the present analyses, they represent important methodological considerations for future research seeking to disentangle morphological advantage from physiological or technical superiority, particularly in developing athlete populations [23,26,28].

Biological maturation and sex-related differences are also known to influence body composition and cardiometabolic capacity. While direct indices of biological maturation were not assessed in the present study, the elite status of the cohort and the use of internationally recognized competitive age categories likely reduced maturational heterogeneity. Nevertheless, age- and sex-specific benchmarking—and, where feasible, maturity-adjusted analyses—remain important considerations when interpreting anthropometry–performance relationships in adolescent rowers [15,41]. An important question for future longitudinal research is whether the observed morphological advantages persist, amplify, or attenuate with continued training and biological maturation, particularly as technical and metabolic adaptations increasingly contribute to performance progression.

From a practical perspective, higher-performing athletes in the present cohort typically exhibited body surface area values approaching or exceeding ~1.90 m^2^ and relative muscle mass values above ~40%, suggesting these ranges as indicative reference benchmarks rather than fixed selection cut-offs. Integrating anthropometric profiling into youth development systems may therefore enhance talent identification by enabling practitioners to distinguish emerging performance potential based on multidimensional morphological signatures. Rather than relying solely on chronological age, sex, or body mass, profile-based approaches may offer a more holistic and developmentally relevant framework for evaluating rowing-specific performance capacity.

Finally, beyond morphology and aerobic capacity, technical and neuromuscular determinants—including lower-limb force–velocity characteristics, stroke length and rate, and rigging configuration—are known to meaningfully influence ergometer output and on-water speed [34,42,43].

**Limitations**

The present study has several limitations that should be considered when interpreting the findings. Its cross-sectional design precludes causal inference and does not allow assessment of longitudinal developmental trajectories in anthropometric characteristics or rowing performance. In addition, the exclusive inclusion of nationally elite junior and adult rowers may limit the generalizability of the results to sub-elite or recreational populations, although this selection enhances the relevance of the findings for high-performance talent identification contexts.

Direct indices of biological maturation, such as years from peak height velocity or maturity offset, were not assessed. While biological maturation is an important consideration in studies involving developing athletes, maturational variability within the present cohort was likely reduced due to the elite status of the participants and the use of internationally recognized competitive age categories.

Body composition was assessed using bioelectrical impedance analysis, which is subject to methodological constraints including sensitivity to hydration status, reliance on device-specific prediction equations, and limited validity in athletic populations with extreme body compositions. Furthermore, rowing performance was evaluated using ergometer-based testing, which does not fully capture the technical, tactical, and environmental demands of on-water competition.

Finally, several potentially relevant confounding variables, such as training volume and intensity, technical rowing proficiency, psychological characteristics, and nutritional status, were not assessed and may have influenced performance outcomes.

## 5. Conclusions

In conclusion, the findings of the present study indicate that rowing ergometer performance in youth athletes reflects multidimensional anthropometric configurations rather than isolated morphological traits. Performance was characterized primarily by the combined contribution of body surface area, relative muscle mass, and segmental body dimensions, underscoring the relevance of integrated anthropometric profiling in the early stages of rowing talent identification.

From a practical perspective, higher-performing athletes in the present cohort typically exhibited body surface area values approaching or exceeding ~1.90 m^2^ and relative muscle mass values above ~40%. These values should be interpreted as indicative reference benchmarks rather than fixed selection thresholds and may assist coaches and practitioners in distinguishing higher- from moderately performing rowers within comparable age and sex categories.

While morphological characteristics represent relatively stable determinants of rowing potential, the cardiorespiratory and metabolic capacities of youth athletes remain highly trainable and can be substantially enhanced through systematic, developmentally appropriate training. Accordingly, training programs should account for age- and sex-specific requirements and prioritize the progressive development of physical and technical competencies most relevant to rowing performance.

At later stages of athlete development—such as squad selection or role assignment within a crew—physiological and performance-related assessments become increasingly important for differentiating between athletes with comparable anthropometric profiles. In such cases, standardized ergometer tests (e.g., 2000 m performance), aerobic capacity indicators, and indices of power–endurance may provide additional, selection-relevant information beyond morphology alone.

Integrating anthropometric profiling with physiological assessment offers a refined, multidimensional framework for talent identification and individualized training design in competitive youth rowing. From a long-term athlete development perspective, such an approach may support the monitoring of maturation-related changes, the optimization of workload progression, and the transition from junior to adult categories. Establishing normative reference profiles across developmental stages may ultimately enhance evidence-based decision-making in athlete selection and align training demands with individual morphological and physiological capacities.

## Figures and Tables

**Table 1:** Differences in anthropometric and performance results of males and females from all nations.

Variables	Female (n = 48) Mean	SD	Male (n = 146) Mean	SD	*p*	Cohen’s d, 95% CI
Sport Age (year)	13.254	4.024	13.953	4.521	0.341	−0.16 [−0.49, 0.17]
Rowing Age (year)	7.396	3.187	8.681	4.134	0.051	−0.33 [−0.66, 0.00]
Height (cm)	171.125	6.327	183.921	6.539	<0.001	−1.97 [−2.35, −1.59]
Body mass (kg)	65.006	6.564	77.710	9.324	<0.001	−1.46 [−1.81, −1.10]
F% (%)	26.040	5.484	14.828	4.744	<0.001	2.13 [1.73, 2.52]
M% (%)	32.755	2.838	42.218	3.231	<0.001	−3.01 [−3.46, −2.56]
BMI (kg/m^2^)	22.183	1.705	23.151	3.411	0.61	−0.31 [−0.64, 0.01]
Sitting height (cm)	89.083	3.683	94.215	3.777	<0.001	−1.37 [−1.72, −1.01]
Arm span (cm)	172.365	7.843	188.605	7.687	<0.001	−2.10 [−2.48, −1.71]
Lower limb length (cm)	100.146	4.976	105.028	5.691	<0.001	−0.88 [−1.22, −0.55]
BSA (m^2^)	1.756	0.112	1.997	0.167	<0.001	−1.55 [−1.91, −1.18]
Age	18.717	3.073	19.332	4.243	0.356	−0.15 [−0.48, 0.17]
Ergo (W)	237.546	30.384	359.453	45.736	<0.001	−2.87 [−3.30, −2.43]

Legend: M% = relative muscle mass, F% = relative fat mass, BSA = body surface area (m^2^), Sport Age indicates total sport participation, and Rowing Age rowing-specific training experience.

**Table 2:** Differences in anthropometric and performance results between age groups and gender.

	JF (n = 27)		JM (n = 71)		AF (n = 21)		AM (n = 75)		*p*
Variables	Mean	SD	Mean	SD	Mean	SD	Mean	SD	
Sport Age	12.542	2.515	11.636	2.669	14.170	5.316	16.147	4.826	AF, AM > JF, JM *
Rowing Age	6.322	1.580	6.638	1.903	8.776	4.132	10.588	4.717	AF, AM > JF, JM *
Age	17.067	0.577	17.031	0.539	20.839	3.654	21.510	5.012	AF, AM > JF, JM * AM > AF *
Height (cm)	170.185	6.045	184.120	6.755	172.333	6.621	183.733	6.368	JM, AM > JF, AF *
body mass (kg)	64.989	7.001	77.289	9.277	65.029	6.126	78.115	9.414	JM, AM > JF, AF *
F% (%)	25.600	4.789	14.016	4.517	26.635	6.385	15.597	5.713	JM, AM > JF, AF *
M% (%)	33.256	3.095	42.242	2.793	32.080	2.356	42.196	3.611	JM, AM > JF, AF *
BMI (kg/m^2^)	22.407	1.741	22.770	2.241	21.895	1.654	23.512	4.216	*p* > 0.05
BSA (m^2^)	1.751	0.117	1.986	0.145	1.763	0.108	2.008	0.186	JM, AM > JF, AF *
Sitting height (cm)	88.741	3.241	94.243	4.016	89.524	4.226	94.189	3.564	JM, AM > JF, AF *
Arm span (cm)	171.333	8.353	188.406	7.711	173.690	7.111	188.791	7.713	JM, AM > JF, AF *
Lower limb length (cm)	99.444	4.635	105.972	6.171	101.048	5.362	104.122	5.067	JM, AM > JF, AF *
Ergo (W)	227.733	32.010	339.994	46.106	250.162	23.246	377.875	37.157	JM, AM > JF, AF * AM > JM *

Legend: JF = junior female, JM = junior male, AF = adult female, AM = adult male. Values are presented as mean ± SD. Group differences reflect significant main effects of age group and/or sex based on two-way ANOVA with Bonferroni-adjusted post hoc comparisons. * = Notations such as “AF, AM > JF, JM” indicate a significant main effect of age, whereas comparisons such as “AM > AF” or “JM > JF” indicate sex-related differences within age categories. *p* < 0.05. Sport Age indicates total sport participation, and Rowing Age rowing-specific training experience.

## Data Availability

The original contributions presented in this study are included in the article. Further inquiries can be directed to the corresponding author.

## Abbreviations

The following abbreviations are used in this manuscript:

BMI	Body mass index (kg/m^2^)
BH	Body height (cm)
BSA	Body surface area (m^2^)
BW	Body weight (kg)
M%	Relative muscle mass
F%	Relative fat mass
